# Merging Data with Modeling: An Example from Fatigue

**DOI:** 10.3390/ma17143383

**Published:** 2024-07-09

**Authors:** D. Gary Harlow

**Affiliations:** Mechanical Engineering and Mechanics, Lehigh University, 27 Memorial Drive W, Bethlehem, PA 18015, USA; dgh0@lehigh.edu

**Keywords:** calibration, model, prediction, sample size, synthesis, uncertainty, validation

## Abstract

It is well known that errors are inevitable in experimental observations, but it is equally unavoidable to eliminate errors in modeling the process leading to the experimental observations. If estimation and prediction are to be done with reasonable accuracy, the accumulated errors must be adequately managed. Research in fatigue is challenging because modeling can be quite complex. Furthermore, experimentation is time-consuming, which frequently yields limited data. Both of these exacerbate the magnitude of the potential error. The purpose of this paper is to demonstrate a procedure that combines modeling with independent experimental data to improve the estimation of the cumulative distribution function (cdf) for fatigue life. Subsequently, the effect of intrinsic error will be minimized. Herein, a simplified fatigue crack growth modeling is used. The data considered are a well-known collection of fatigue lives for an aluminum alloy. For lower applied stresses, the fatigue lives can range over an order of magnitude and up to 10^7^ cycles. For larger applied stresses, the scatter in the lives is considerably reduced. Consequently, modeling must encompass a variety of conditions. The primary conclusion of the effort is that merging independent experimental data with a reasonably acceptable model vastly improves the accuracy of the calibrated cdfs for fatigue life, given the loading conditions. This allows for improved life estimation and prediction. For the aluminum data, the calibrated cdfs are shown to be quite good by using statistical goodness-of-fit tests, stress-life (S-N) analysis, and confidence bounds estimated using the mean square error (*MSE*) method. A short investigation into the effect of sample size is also included. Thus, the proposed methodology is warranted.

## 1. Introduction

Error analysis has been employed for quite some time, especially for complex engineering problems. Typical issues associated with error analysis are determining how errors combine, how errors propagate, or, more importantly, how errors are mitigated. Uncertainty can occur either by systematic or random errors. Frequently, propagation of uncorrelated errors is assessed by using the square root of the sum of the squares of the errors. The difficulty in this type of analysis is determining how many contributors to the total uncertainty need to be considered. Useful presentations of error analysis are contained in references [1,2,3,4,5]. An article that uses error analysis as a teaching tool is [6]. The content is well presented, and there is an excellent reference section. A more recent journal article that uses error analysis is [7]. The authors provide a concise review of the basic approaches for error analysis: the reliability state function, the first-order second-moment method, the response surface method, and sensitivity analysis of errors. For the mechanical system that they considered, they were successful with their approach. They also provide a valuable reference list. It goes without saying that error analysis depends on the accuracy of the model and the data. The amount of uncertainty in each variable in the model increases the overall uncertainty. As more sources of uncertainty are considered, the overall uncertainty must increase. Without some control on uncertainty, it can become so large that the results are overshadowed by the accumulated error.

The approach to be demonstrated below is a combination of scientifically or physically based modeling with adjustments made by strategically fusing an independent set of experimental data. The method was first developed for modeling the yield strength for an aircraft engine alloy [8]. Extensive mechanistic and structural materials modeling was employed to estimate the yield strength. Due to modeling error, even though the scientific model predictions were detailed and thorough, they did not adequately match the rich databases that were to be used for validation for the yield strength prediction. The model was subsequently calibrated by using the additional yield strength data. The result was that, for this application, the calibrated yield strength was excellent for estimation and prediction. A major reason for the success in this problem is that the variability in the simulated model results and the experimental data for the yield strengths are reasonably small. Furthermore, yield strength is time-independent.

Fatigue, obviously, is time-dependent, and, consequently, life data tend to have greater variability. The proposed methodology was applied to data from a very high cycle fatigue application for a steel [9]. The selected data were primarily for three distinct cases. One case, for relatively high applied load, exhibited failure induced by surface abnormalities only. The amount of scatter was comparatively small. For another case, with moderate applied load, the failures were primarily induced by surface flaws, but there were a few failures that initiated at subsurface inclusions. In this case, the scatter in fatigue lives was almost four orders of magnitude. The final example was for a smaller applied load for which about half of the failures were surface-induced and the remainder were internally induced. The scatter was about three orders of magnitude. This application included uncertainty from a variety of inputs, and the bimodal behavior is an added complication. Thus, a mechanistic model that characterizes the complexities is necessary because inadequacies or oversights are contributors to uncertainty. As modeling complexity increases, the amount of extra data needed for calibration also increases. The overriding conclusion from this work is that calibration of a viable model with sufficient data improves reliability estimation and prediction. The technique seems to be appropriate and warranted.

Based on the success of the above examples, the purpose of this work is to investigate the applicability of the methodology for two other sets of fatigue data. The integration of fatigue life data with a mechanistic model is investigated for data given in Shimokawa and Hamaguchi [10]. This is a detailed and reputable set of data. These data have been used by others; one recent example is [11]. Again, the strategy is to incorporate suitable additional fatigue data with mechanistic modeling to overcome inherent error and to improve subsequent reliability estimations and predictions with statistical confidence.

## 2. 2024-T4 Aluminum Alloy

Shimokawa and Hamaguchi [10] established a rather large collection of fatigue data for 2024-T4 Aluminum Alloy (AA). The specimens were subjected to constant amplitude loading. They conducted tests on rectangular specimens that were 110 mm long, 52 mm wide, and 1 mm thick. The principle focus of their investigation was the effect of different types of notches or holes on fatigue life. The analyses below utilize two of their experimental programs.

### 2.1. Center Cut Circular Hole

These samples had a center cut circular hole of radius 5 mm. The fatigue data for this condition are summarized in Table 1, which is reproduced from [10] for completeness. Note that Δσ is the applied stress amplitude, and, for each Δσ, *n* is the sample size, $\bar{x}$ is the sample average, *s* is the sample standard deviation, and *cv* is the sample coefficient of variation. For the eight different values of Δσ, there were a total of 222 fatigue tests conducted. Clearly, the statistical behavior is different when Δσ is 157 MPa and greater compared to applied loads less than that. For Δσ that replicate ordinary operations, fatigue lives are usually longer with greater variability. Thus, modeling requires special consideration.

**Figure 1:**
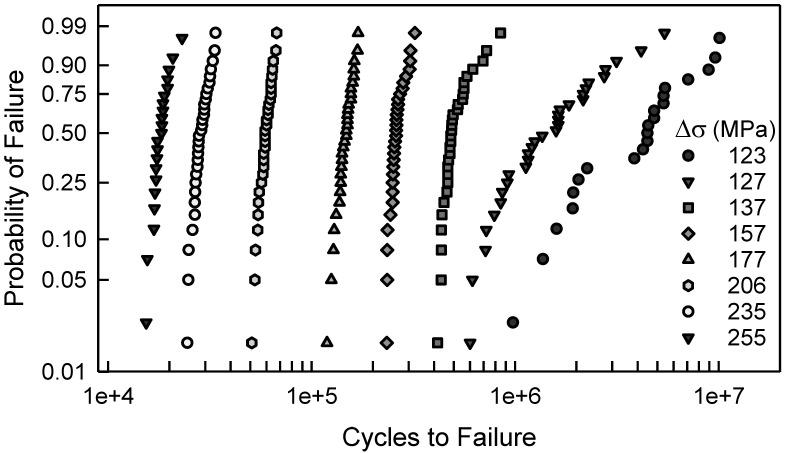
Fatigue failure data for 2024-T4 AA given Δσ; specimens with a center cut circular hole [10].

**Figure 2:**
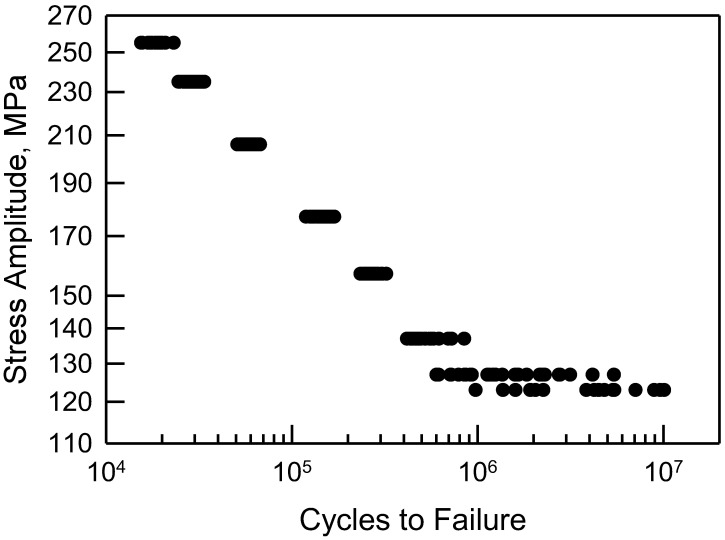
S-N data for 2024-T4 AA; specimens with a center cut circular hole [10].

The fatigue failure data for the 2024-T4 AA specimens with a center cut circular hole are shown in Figure 1. The data are plotted on two-parameter Weibull probability paper. By observation, a two-parameter Weibull cumulative distribution function (cdf) is acceptable for Δσ greater than or equal to 177 MPa. For Δσ less than 177 MPa, a two-parameter Weibull cdf is not acceptable because the tails of the data deviate too much from linearity. In other words, the Anderson–Darling (AD) goodness-of-fit test indicates that a two-parameter Weibull cdf is not acceptable for these data. Possibly, a three-parameter Weibull cdf might be a better choice; however, the AD test implies that the tails of the data for Δσ, equal to 127 and 137 MPa, are sufficiently different that three-parameter Weibull cdfs are not acceptable. A log-normal cdf was proposed in [10]. It was indicated in that paper that the log-normal cdf was a better choice than a two-parameter Weibull cdf. When Δσ equals 127 or 137 MPa, a log-normal cdf is not acceptable, according to the AD test. All of the above discussion is solely statistical modeling which is empirical, and the selection of a cdf is grounded in pragmatism. It is also appropriate to show the fatigue data in a stress-life (S-N) format. Figure 2 displays the S-N data for the specimens with a center cut circular hole. As mentioned above, it is clear graphically that the scatter is nearly the same for each Δσ above 150 MPa, but below 150 MPa, the scatter is significantly greater. For the two smallest values of Δσ, the scatter exceeds an order of magnitude.

### 2.2. Center Cut Notch

Another series of fatigue experiments for 2024-T4 AA that were included in [10] were for the same rectangular specimens but with a different notch design. These specimens had a center cut notch 10 mm long with a maximum center width of 3 mm tapered down to a tip of radius 0.25 mm. The loading was perpendicular to the notch design. The fatigue lives are summarized in Table 2. There were nine different values for Δσ, for a total of 252 tests for this specimen type. For Δσ greater than or equal to 147 MPa, the scatter in fatigue lives is about the same; however, when Δσ is less than 147 MPa, the scatter increases as Δσ decreases. Notice the asterisk for $\bar{x}$, *s*, and *cv* when Δσ is 64 MPa. The reason is that the three maximum fatigue data for this case are censored. Thus, an estimate for the mean and standard deviation cannot be computed by simple averaging, as with the other values for Δσ. An excellent nonparametric estimate for the mean and standard deviation can be obtained by using the Kaplan–Meier estimator for the empirical distribution function. A well-developed presentation of the Kaplan–Meier methodology can be found in [12].

**Figure 3:**
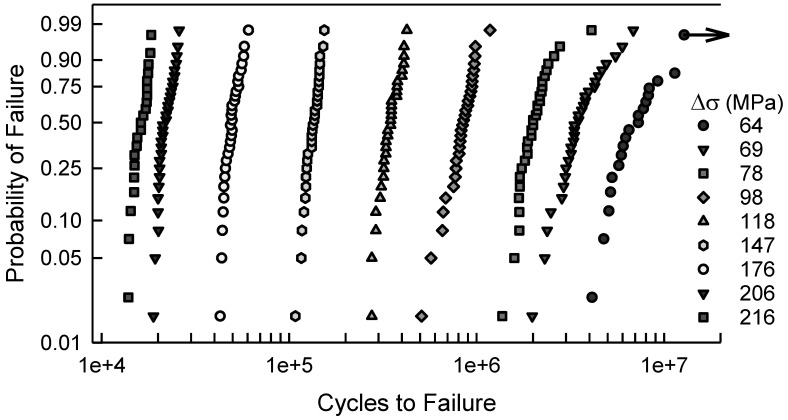
Fatigue failure data for 2024-T4 AA given Δσ; specimens with a center cut notch [10].

**Figure 4:**
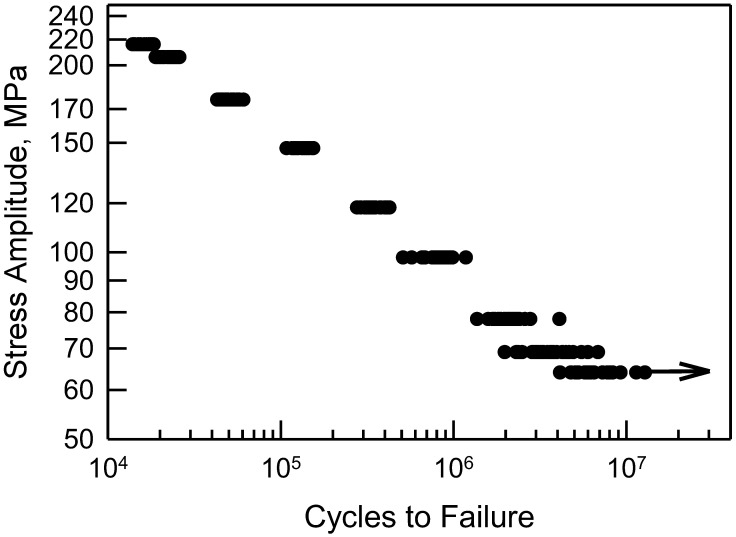
S-N data for 2024-T4 AA; specimens with a center cut notch [10].

For the notched specimens, the fatigue failure data are shown in Figure 3. There are similarities to the data in Figure 1. For the six largest values of Δσ, a two-parameter Weibull cdf is acceptable; however, for the other three conditions, it would not be acceptable. The scatter in the data coupled with the curvature makes a statistical fit more challenging. Furthermore, when Δσ is 64 MPa, there are three identical data that were censored, as indicated by the arrow in Figure 3. Thus, the censoring has a significant effect for fitting a cdf for these data. Again, empirically selecting a suitable cdf is nontrivial, and it appears that more involved analysis may be needed. Likewise, Figure 4 is the S-N diagram for specimens with a notch. When Δσ exceeds 100 MPa, the scatter is similar, but for less than 100 MPa, the scatter increases as Δσ decreases. Again, the arrow indicates that there are three similar data that were censored.

## 3. A Fatigue Crack Growth Model for the 2024-T4 AA Data

The selection of an acceptable mechanistic model for any fatigue problem is difficult. This is equally true for the two examples considered herein. A nontrivial reason for this is that the experiments reported in [10] were conducted about four decades ago. Nevertheless, a simplified fatigue crack growth model is proposed. Given a crack length of *a* for *N* cycles, the crack growth rate *da/dN* is assumed to be characterized by the following equation:

(1) $$\frac{da}{dN}=C{\left(\Delta K-\Delta K_{th}\right)}^{\rho },$$

where Δ*K* is the driving force and Δ*K_th_* is the threshold. The materials constants for 2024-T4 AA are *C* and ρ. For the 2024-T4 AA considered, ρ is assumed to be 3.33.

### 3.1. Center Cut Circular Hole

Failure is assumed to be caused by a semi-circular surface crack that transitions into a through-the-thickness crack. Thus, Δ*K* differs for the two regimes. The driving force for a surface crack (sc) Δ*K_sc_* is assumed to be the following:

(2) $$\Delta K_{sc}=(2.24/\pi )k_{t}\Delta \sigma \sqrt{\pi a},$$

where 2.24/π is the geometric factor for a semi-circular crack in an infinite plate, and *k_t_* is the stress concentration factor for the hole. Using Figure 2.59 in [13] for an estimated value for the stress concentration factor for the test specimens, *k_t_* is 2.5. Similarly, the driving force for a through-the-thickness crack (tc) Δ*K_tc_* is

(3) $$\Delta K_{tc}=F_{tc}(a/r_{o})\Delta \sigma \sqrt{\pi a},$$

where *r_o_* is the radius of the hole. Numerical values for *F_tc_*(*a*/*r_o_*) for an infinite plate under uniaxial tension containing a circular hole with a single through crack emanating from the hole perpendicular to the loading axis can be fit empirically, to within graphical resolution, by the following function:

(4) $$F_{tc}(a/r_{o})=0.681+\{0.865/[(a/r_{o})+0.324]\};$$

See reference [14]. Equations (3) and (4) were used for simplicity and computational convenience.

The fatigue life *N_f_* is the sum of the cycles needed for the surface crack growth *N_sc_* and the through-the-thickness crack growth *N_tc_*, i.e.,

(5) $$N_{f}=N_{sc}+N_{tc}=\int_{a_{o}}^{a_{tc}}\frac{da}{C{\left(\Delta K_{sc}-\Delta K_{th}\right)}^{n}}+\int_{a_{tc}}^{a_{f}}\frac{da}{C{\left(\Delta K_{tc}-\Delta K_{th}\right)}^{n}},$$

where *a_o_* is the initial damage size, *a_tc_* is the crack size at which the surface crack transitions into a through-the-thickness crack, and *a_f_* is the final crack size. The integrals in Equation (5) are clearly from Equation (1). With Δ*K_sc_* given in Equation (2), the first integral can be explicitly integrated. With Δ*K_tc_* defined by Equation (3), the second has to be integrated numerically. It is assumed that a surface crack transitions into a through-the-thickness crack at *a_tc_*, which is the solution of

(6) $$F_{tc}(a_{tc}/r_{o})=(2.24/\pi )k_{t},$$

i.e.,

(7) $$a_{tc}=r_{o}\left[\frac{0.865}{(2.24k_{t}/\pi )-0.681}-0.324\right].$$

For the specimens under consideration, *r_o_* is 5 mm, which implies that *a_tc_* is 2.31 mm. Since the width of the specimen is 52 mm, *a_f_* is set to be 21 mm.

The variables *C*, Δ*K_th_*, and *a_o_* are assumed to be random variables (rvs) that characterize the variability in the microstructural properties of the material. They are also assumed to be independent of the loading and time. A three-parameter Weibull cdf has been used frequently to represent material properties, and it is used for these rvs. The form used herein is given by

(8) $$F(x)=1-\exp\{-{\left[(x-\gamma )/\beta \right]}^{\alpha }\},\;\;x\geq \gamma$$

where α is the shape parameter, β is the scale parameter, and γ is the minimum. An important property of Equation (8) is the mean μ, which is

(9) μ=γ+βΓ(1+1/α),

where Γ (∙) is the Gamma function. Another significant characteristic is the *cv*, which is

(10) $$cv=\frac{\beta \sqrt{\Gamma (1+2/\alpha )-\Gamma^{2}(1+1/\alpha )}}{\gamma +\beta \Gamma (1+1/\alpha )}.$$

The estimated values for the parameters for the rvs are based on a conglomeration of data for 2024-T3; see [15,16,17,18,19,20]. It is assumed that the material properties are sufficiently close for those of 2024-T4 that they can be used for the ensuing analyses. Table 3 contains the parameter values for the rvs used for the subsequent computations.

**Figure 5:**
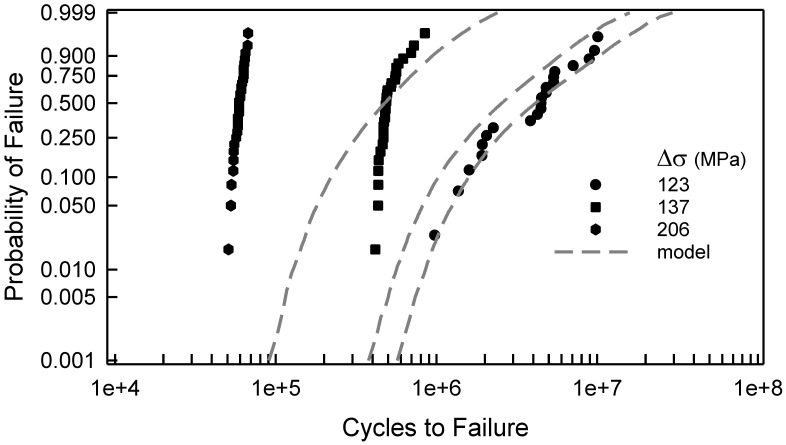
Selected fatigue failure data for 2024-T4 AA specimens with a center cut circular hole [10], and the corresponding simulated model for the given Δσ.

Figure 5 shows the fatigue failure data for Δσ equal to 123, 137, and 206 MPa, which are also in Figure 1. The dashed lines are the simulated model cdfs developed above, which is entirely independent of the fatigue lives. The model is quite good when Δσ is 123 MPa. When Δσ is 137 or 206, the model is not suitable at all. In fact, the model and the data have a maximum deviation of almost an order of magnitude when Δσ is 206 MPa. For the other values of Δσ shown in Figure 1, the model is likewise not appropriate. Consequently, an alternative approach is required.

### 3.2. Center Cut Notch

For this case, the stress concentration factor used in [10] is 3.8, and that is assumed for the ensuing computations as well. The failure, again, is assumed to be caused by a semi-circular surface crack that emanates from the notch. Since the through-the-thickness portion of the crack growth was relatively insignificant for the center cut circular hole computation, it has been omitted for this case. Thus, Δ*K* is assumed to have the same form as Equation (2). The rvs *C* and Δ*K_th_* are assumed to have the same cdfs as above because they are characteristic of the material properties. For *a_o_*, however, the surface area from which a crack emanates for the center cut circle specimens is about 20 times greater than that for the center cut notch specimens. Consequently, the cdf for *a_o_* is adjusted. The mean is increased to 19.9 × 10^−6^ m and the *cv* is reduced to 10.4%. As the surface area under high stress decreases, the critical size for the crack initiation increases, but with fewer such sites in the field. Clearly, this needs to be verified.

**Figure 6:**
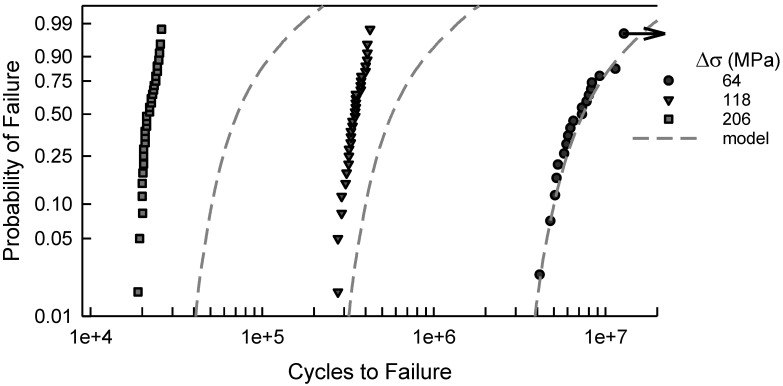
Selected fatigue failure data for 2024-T4 AA specimens with a center cut notch [10], and the corresponding simulated model for the given Δσ.

In Figure 6, the fatigue failure data shown are for Δσ equal to 64, 118, and 206 MPa. These data are also part of Figure 3. Again, the dashed lines are the cdfs computed by simulating the model, which is an independent computation from the experimental fatigue lives shown. When Δσ is 64 MPa, the model is graphically quite good. Recall that the arrow indicates censored data. For the other two cases shown, Δσ is 118 or 206, the model represents the data quite poorly. Likewise, for the other values of Δσ shown in Figure 3, the model is unacceptable. As indicated with the center cut circular hole data, a different tactic is needed.

## 4. Model Calibration for Fatigue Life Analysis

The modeling for the cdfs shown in Figure 5 and Figure 6 is excellent for the smallest applied load, when Δσ is 123 MPa for the center cut circular hole case and when Δσ is 64 MPa for the center cut notch condition; however, for the others, they are poor representations of the experimental data. Fortunately, these data for each Δσ are independent of the modeling, and they are available to augment the modeling results. The proposed approach to control the difference between the fatigue data and the model simulations is a straightforward empirical calibration. For each given value of the applied stress Δσ, let *N_i_* be an experimental fatigue life out of a total of *n*, and similarly, let *Y_j_* be one of the *m* simulated values for the model. Because the magnitude of fatigue lives for the data and model simulations are so large, and because they frequently exhibit substantial scatter, they are transformed initially using the natural logarithm. That is, let *LN_i_* and *LY_j_* be *ln*(*N_i_*) and *ln*(*Y_j_*), respectively. The transformation *LZ_j_* that is applied to *LY_j_* consists of a rotation and translation, so that the sample averages and sample standard deviations of the *LN_i_* and *LZ_j_* collections are identical. This is accomplished by the following equations:

(11) $$LZ_{j}=aLY_{j}+b,$$

where

(12) $$a=\frac{s_{LN}}{s_{LY}}\;\mathrm{and}\;b=\bar{LN}-\frac{s_{LN}}{s_{LY}}\bar{LY},$$

where $\bar{LN}$ and $\bar{LY}$ are the sample averages, and *s_LN_* and *s_LY_* are the sample standard deviations of $\left\{LN_{i}:1\leq i\leq n\right\}$ and $\left\{LY_{i}:1\leq i\leq m\right\}$, respectively. To return to actual cycles, the *LZ_j_* values are transformed by applying the exponential function.

### 4.1. Center Cut Circular Hole

**Figure 7:**
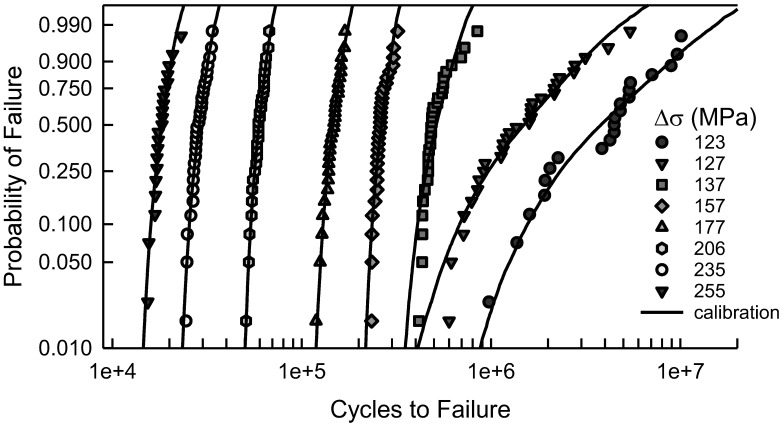
Fatigue failure data for 2024-T4 AA specimens with a center cut circular hole [10], and the corresponding calibrated cdfs for the given Δσ.

Figure 7 shows the fatigue data for the center cut circular hole specimens, which are also in Figure 1. In addition, the solid lines are the calibrated cdfs as described above. Visually, all of the calibrated cdfs characterize the data quite well. A comparison of the model cdf in Figure 5 with the calibrated cdf in Figure 7 when Δσ is 123 MPa indicates very little difference. Undoubtedly, if the model is accurate, there is little need for any calibration. When Δσ is 137 MPa or 206 MPa, however, the contrast between the model cdfs and the calibrated cdfs is striking. It clearly demonstrates the need for the translation and rotation in Equation (11). The Kolmogorov–Smirnov (KS) and AD goodness of fit tests were applied to validate the quality of the calibrated cdfs. The largest KS test statistic for the eight different values of Δσ is 0.18, which indicates that the calibrated cdf is acceptable for each value of Δσ for any significance level less than 0.20. The KS test primarily reflects the behavior of the central region of the data. The AD test implies that the cdfs are acceptable for the same significance level for each Δσ except 137 and 157 MPa. These two cdfs are not acceptable, according to the AD test. The reason for this observation is that the AD test describes the behavior in the tails of the cdf. This is apparent in Figure 7 because the tails are quite distinct from the fatigue data. In both cases, however, the cdfs are on the conservative side of the data, and would serve as suitable cdfs for prediction. Therefore, the calibrated cdfs are acceptable representations of the fatigue data. Because the calibrated cdfs are a combination of basic mechanistic modeling and experimental fatigue data, they are appropriate for estimation and prediction beyond the data range, especially for applied loads that represent typical operating conditions.

**Figure 8:**
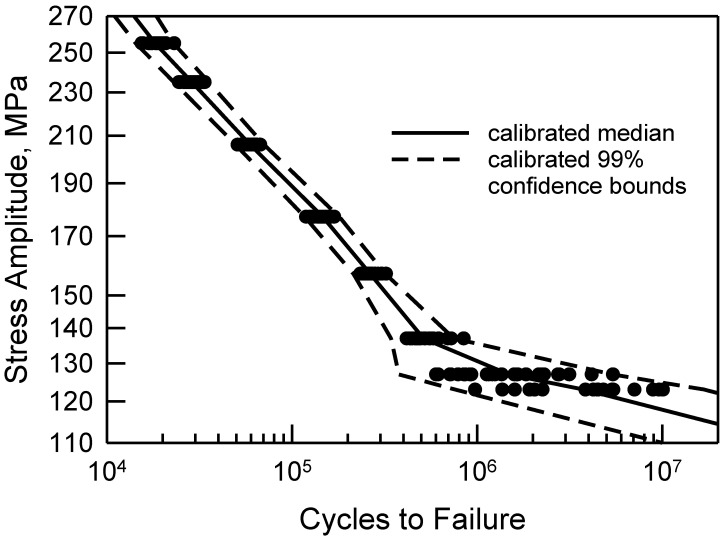
S-N data for 2024-T4 AA; specimens with a center cut circular hole [10], and estimated median and 99% confidence bounds from the calibrated cdfs.

Another way in which to assess the validity of the calibrated cdfs is to consider the S-N behavior. Consider Figure 8, which is a reproduction of Figure 2 with estimated percentile lines added. These percentiles are taken directly from the calibrated cdfs shown in Figure 7. The solid line consists of the estimated medians. Because the calibrated cdfs are excellent representations of the central portion of the data, the estimated medians are also quite good. The dashed lines are the estimated 99% confidence bounds. The upper bound is the estimated 99.5 percentile and the lower bound is the 0.5 percentile computed from the calibrated cdfs. All the data lie between the bounds, and the bounds are very tight. Using the calibrated cdfs provides an excellent characterization of the fatigue data. As a final comment, it should be noted that the sharp corner on the lower bound when Δσ is 127 MPa corresponds to the difference in the calibrated cdf and the data in the lower tail in Figure 7. Because the calibrated cdf is conservative, the lower bound is appropriate.

### 4.2. Center Cut Notch

**Figure 9:**
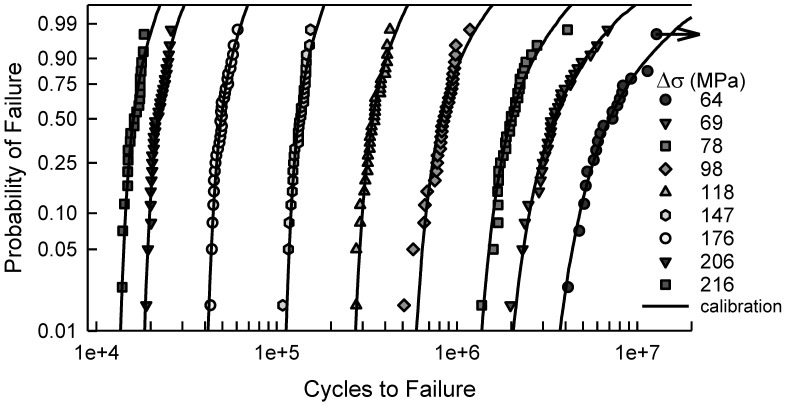
Fatigue failure data for 2024-T4 AA specimens with a center cut notch [10], and the corresponding calibrated cdfs for the given Δσ.

As with the above example, Figure 9 contains the fatigue data for the center cut notch specimens, which were also shown in Figure 3, and the calibrated cdfs for each value of Δσ. Recall that the arrow in Figure 9 indicates that there are three censored data. Graphically, the calibrated cdfs characterize the data quite well. Except for the smallest data when Δσ is 98 MPa and the largest data when Δσ is 78 MPa, the data are close to the calibrated cdfs. As with the center cut circular hole case when Δσ is the smallest, i.e., 123 MPa, when Δσ is 64 MPa, the difference between the model cdf and the calibrated cdf is very small. To further assess the goodness of fit of the calibrated cdfs, the KS and AD tests were used. The KS test indicates that all of the calibrated cdfs are acceptable for any significance less than 0.20. The AD test infers that the calibrated cdfs are acceptable for any significance value less than 0.20, except when Δσ is 98 MPa, 147 MPa, and 216 MPa. The AD test implies that the calibrated cdf is not acceptable when Δσ is 98 MPa. This is clearly seen in Figure 9 because the tails are not very close to the calibrated cdf. Finally, the calibrated cdfs for Δσ equal to 147 MPa and 216 MPa are acceptable for a significance of 0.05. All things considered, the calibrated cdfs are acceptable as estimates for the center cut notch fatigue data, except for when Δσ is 98 MPa.

Before continuing, recall that because of the censored data when Δσ equals 64 MPa, the sample average and standard deviation were estimated by using the Kaplan–Meier methodology [12]. They are recorded in Table 2. These estimates were used in the calibration; see Equation (12). The inference is that the proposed calibration approach is also suitable when censored data are part of the results. In fact, the methodology requires no modification as long as the sample average and standard deviation can be suitably estimated.

**Figure 10:**
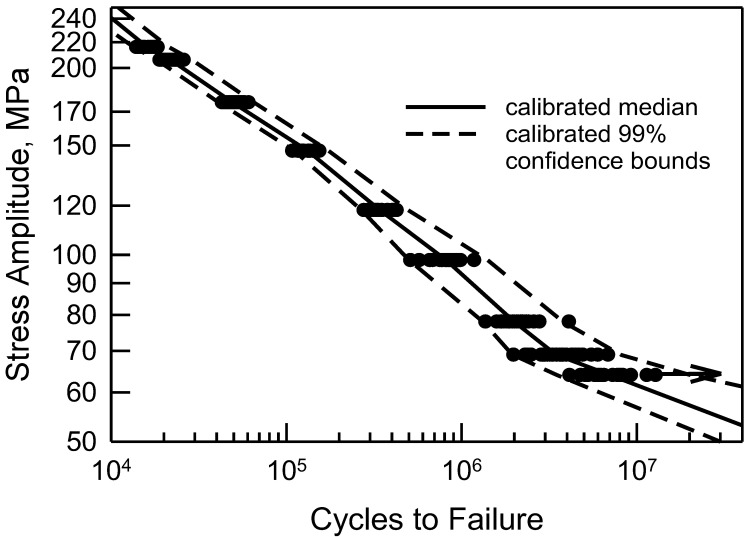
S-N data for 2024-T4 AA; specimens with a center cut notch [10], and estimated median and 99% confidence bounds from the calibrated cdfs.

Figure 10 shows the S-N data from Figure 4 with the estimated percentile lines. As before, the median behavior is the solid line, and the 99% confidence bounds are the dashed lines; all of these are obtained from the calibrated cdfs shown in Figure 9. The estimated medians are excellent. The confidence bounds characterize the data well. Not only are they close to the data, but they also reflect the scatter for each given value of Δσ. For the censored data, it is conceivable that the actual life is outside the confidence bounds. Even if this were the case, the bounds are excellent because they are conservative.

## 5. Mean Square Error Analysis

Mean Square Error (*MSE*) analysis is a well-known methodology to assess the validity of an estimation. The error *e_i_* is the difference between the calibrated cdf and the fatigue data. The *MSE* is given by

(13) $$MSE=\frac{1}{n}\sum_{i=1}^{n}e_{i}^{2}.$$

Approximating confidence bounds with the *MSE* is typically done by using the square root of the *MSE* in Equation (13). Let σ*_MSE_* be the square root of the *MSE*, which can be taken as an estimate for the standard deviation. For unbiased error distributions, the standard error is equivalent to σ*_MSE_*; see reference [21]. Additional information for the *MSE* can be found in [22]. When *e_i_* is epitomized by a normal cdf, 95% confidence bounds are estimated by adding and subtracting 2σ*_MSE_* from the calibrated cdf.

**Figure 11:**
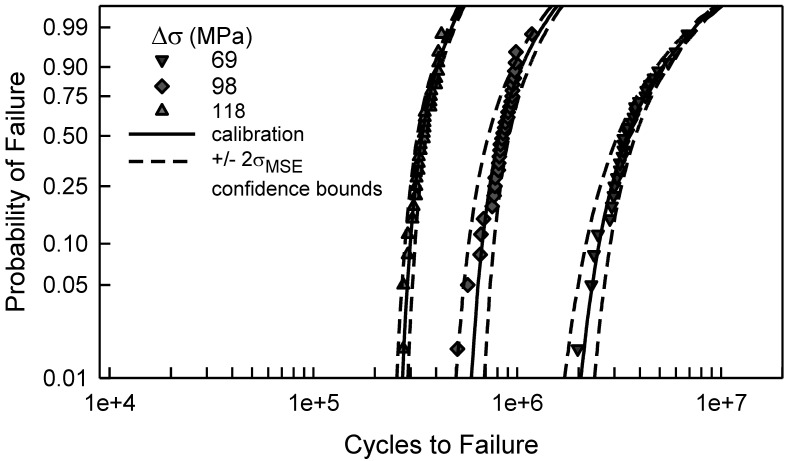
Fatigue failure data for 2024-T4 AA specimens with a center cut notch [10] for selected values of Δσ, the corresponding calibrated cdfs, and *MSE* confidence bounds.

Figure 11 shows the fatigue data for three values of Δσ for the center cut notch specimens along with the calibrated cdfs from Figure 9. The three examples shown represent the range of accuracy for the calibrated cdfs. The dashed lines for each case are the estimated 95% *MSE* confidence bounds. Clearly, the bounds encompass the data in each case. The widths of the bounds are dependent on the accuracy of the calibrated cdf. When Δσ is 118 MPa, the calibrated cdf is an excellent approximation for the data. The average error is only 970 cycles and the corresponding σ*_MSE_* is 8600 cycles. For this case, ±2σ*_MSE_* is only about 5% of the median behavior. Consequently, the bounds reflect the data extremely well. The calibrated cdf is not as close to the data when Δσ is 98 MPa, especially in the lower tail. Here, the average error is 3100 cycles, the corresponding σ*_MSE_* is 47,500 cycles, and ±2σ*_MSE_* is about 12% of the median. Also, the difference between the calibrated cdf and the lower confidence bound is the same as that between the calibrated cdf and the smallest fatigue data. This difference is basically 2σ*_MSE_*. For the center cut notch specimens, the *MSE* confidence bounds are very good for each Δσ with a complete set of fatigue data, including Δσ equal to 98 MPa. The *MSE* analysis is another validation of the proposed methodology.

The *MSE* analysis for confidence bounds for the center cut circular hole data is essentially the same. When Δσ is greater than 157 MPa, the calibrated cdfs are excellent fits to the fatigue data; see Figure 7. For these four, the *MSE* confidence bounds are very tight and envelop all the data, like the example when Δσ is 118 MPa in Figure 11. For Δσ equal to 137 MPa, the *MSE* confidence bounds contain the data, but they are wider because of the deviation in the tails of the cdf. The σ*_MSE_* is 43,000 cycles, and ±2σ*_MSE_* is about 17% of the median. Similarly, when Δσ is 157 MPa, the *MSE* bounds encompass the data and are rather tight because σ*_MSE_* is only 7200 cycles, and ±2σ*_MSE_* is only 5% of the median. For the remaining two values of Δσ, the *MSE* is not acceptable because the error is so large in magnitude. For Δσ equal to 127 MPa, the error between the minimum data and the calibrated cdf is over 150,000 cycles. At the maximum data, the error is over 1,050,000 cycles. Altogether, the average error is 52,000 cycles and σ*_MSE_* is 340,000 cycles. When Δσ is 123 MPa, there are three data points near the median where *e_i_* is quite large. The *MSE* analysis is not as robust for the center cut circular hole; nevertheless, it lends credibility to the proposed methodology.

## 6. Sample Size for Calibration

In experimental work, the overriding issue is the number of tests required to adequately characterize the property being investigated. One of the best and most complete professional guidelines for material properties that indicates the acceptable sample size for a qualified experimental program is MMPDS [23], which is a scientifically developed procedure for metallic materials to assess experimental and design data so that they are acceptable for certification. MMPDS is a joint effort of government agencies and industrial, educational, and international aerospace organizations. Specifically, chapter 9 is related to statistical analysis. In Section 9.9.1.1, the comment is made that for fatigue experimentation subjected to load-controlled conditions, each load should include at least six observations to failure. While this is a good rule of thumb, it may not be sufficient to fully characterize the scatter for a given load condition.

**Figure 12:**
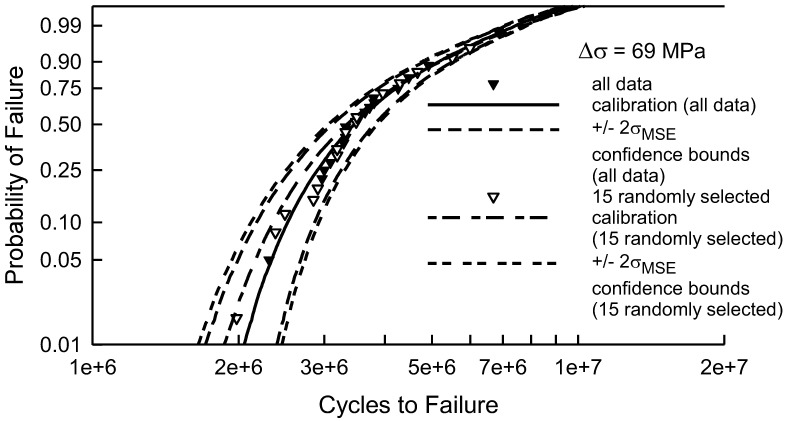
Fatigue failure data for 2024-T4 AA specimens with a center cut notch [10] for Δσ equal to 69 MPa. The calibrated cdfs and *MSE* confidence bounds using all 30 data points and 15 randomly selected data points are shown.

As an example, consider the center cut notch when Δσ is 69 MPa, which is shown in Figure 3, Figure 9 and Figure 11. This example is chosen because there is substantial scatter in the data and the calibrated cdf is an excellent fit. The sample size is 30. The main purpose of this effort was to demonstrate that the calibration method is effective and warranted. The query is whether or not less than 30 data points would have been just as effective for the calibration. Figure 12 shows the fatigue lives when Δσ equals 69 MPa, the calibrated cdf, and the *MSE* confidence bounds, which were also shown in Figure 11. The only difference is that the axis for the cycles has been expanded for the graph in Figure 12. Arbitrarily, 15 out of the 30 data points were randomly selected and used to calibrate the cdf. The white data are the ones that were randomly chosen. The corresponding calibrated cdf using just the 15 randomly selected data points is represented by the short-long dashed line. Graphically, it is reasonably similar to the cdf calibrated using all 30 data points; however, there is some deviation in the lower tail. In fact, the KS and AD tests, comparing the entire sample with the augmented cdf, indicate that it is acceptable for any level of significance less than 0.2. The *MSE* confidence bounds, shown as the short-short dashed lines, are a bit wider, but not overly so. Thus, a sample size of only 15 may have been acceptable. Alas, caution must be exercised because the random sample shown is excellent because the 15 data points are widely distributed over the entire sample of 30. Due to randomness, the 15 selected data points could have primarily reflected the upper tail, which would not have adequately served for calibration. Further analysis is required prior to making a definitive statement about the sample size.

To add a bit more understanding about the required sample size for quality calibration, a random selection from the 30 fatigue data points for Δσ equal to 69 MPa was repeated 1000 times. The size of the random sample was 10, 15, or 20. It should be noted that the total number of ways to select 10 or 20 data points from 30 is over 30 million, and the number of ways to choose 15 out of 30 is over 115 million. Thus, repeating the calibrations 1000 times will not lead to duplications. As expected, when only 10 data points are used for the calibration the ensuing cdf may not be acceptable. Out of the different attempts, the KS test implied that 4.9% would be unacceptable. Of the remainder, 72.6% would be acceptable for any significance below 0.2, and the rest would be acceptable with smaller a significance. The AD test was more severe because 53.0% of the calibrated cdfs were unacceptable, and only 20.7% were acceptable with a significance of 0.2. If 15 data points are used to calibrate the cdf, the results improve. Less than 1% are unacceptable, according to the KS test, but 31% are still unacceptable using the AD test. Using 20 randomly selected data points for the calibration improves the results considerably. The KS test indicates that 100% of the cdfs are acceptable with any significance less than 0.2. Because the tail behavior is more challenging, the AD test yields 11.0% that are unacceptable, and 60.9% that are acceptable with a significance less than 0.2, and the remaining attempts are acceptable with a smaller level of significance.

Certainly, the more data that are available, the better the calibration will be. The scatter in the data is not that large when Δσ is 69 MPa. Thus, fewer data may be sufficient for the calibration. In fact, 25 to 30 data points seems to be appropriate. When there is more scatter in the data, there may need to be more data in order to achieve an acceptable calibration. For example, the lower tail of the data when Δσ equals to 98 MPa is sufficiently different from the calibrated cdf that additional data would be helpful. It is difficult, a priori, to select an appropriate sample size for fatigue testing, but 30 tests for each loading condition is an excellent beginning.

## 7. Results and Discussion

The purpose of this effort was to demonstrate the validity and value of calibrating a cdf for fatigue life with independent experimental data. The cdf is generated from a probabilistic fatigue crack growth model using standard simulation methods. Even though the model is somewhat simplistic, the proposed methodology yields convincing results. The fatigue data considered were taken from [10]. Two different types of specimens, based on different center cut features, were used in the analysis. One set of experiments were conducted with specimens with a center cut circular hole, and the other set used a center cut notch design. There were eight different values for the stress amplitude Δσ for the center cut circular hole specimens, and nine different ones for the center cut notch specimens. An extremely significant feature of these data sets is that the amount of data for each value of Δσ is noteworthy. All things considered, the methodology produces excellent results for estimation and prediction of the fatigue behavior. A primary motive for this process is to improve the characterization and accuracy of the cdf for fatigue life given Δσ. The calibration of the model cdf with data drastically improves the estimation because the uncertainty is controlled empirically. Certainly, as the modeling is improved, the overall accuracy is likewise better, and the reliance on the data for the calibration is diminished. This is illustrated in Figure 7 when Δσ is 123 MPa, and in Figure 9 when Δσ is 64 MPa.

For the fatigue model, it was assumed that three rvs and their associated cdfs were sufficient to capture the majority of the variability. Even so, it was shown that most of the simulated cdfs were not an accurate characterization of the fatigue life. In fact, some cdfs differed from the corresponding data by almost an order of magnitude. Further improvements in modeling could alleviate these discrepancies. Even for cdfs when the scatter in the data was relatively small, there were significant differences.

The proposed calibration method was demonstrated to be quite useful for the two different types of specimens and the multiple values of Δσ for each. The validation for the approach was strongly established for all but three of the values of Δσ; however, even those three were well characterized by the S-N behavior. Nevertheless, the fatigue life model could be improved, which would lead to even more accurate calibrations. From this effort, the proposed methodology appears to be warranted. The approach should be implemented for additional applications to determine its full capability.

A few final comments are in order regarding the sample size needed for the calibration. For fatigue experimentation, especially for critical load bearing components, 30 tests for the key loading conditions is an excellent rule of thumb. If an accurate mechanical model for fatigue can be established, then possibly as few as 15 tests may be sufficient. The sample size is intimately related to the amount of scatter in the data. Data with large scatter will necessarily require more experiments for the calibration.

## Figures and Tables

**Table 1:** Summary of fatigue data for 2024-T4 specimens with a circular notch [10].

Stress Amplitude, Δσ (MPa)	Sample Size, *n*	Median Life	Sample Average, $\bar{x}$	Sample Standard Deviation, *s*	Sample Coefficient of Variation, *cv* (%)
255	21	18,500	18,200	1760	9.6
235	30	29,100	28,700	2500	8.7
206	30	59,300	59,400	4230	7.1
177	30	144,200	146,000	12,600	8.6
157	30	251,700	264,000	22,600	8.6
137	30	469,100	519,000	96,200	18.5
127	30	1,424,700	1,710,000	1,090,000	63.8
123	21	4,401,800	4,530,000	2,660,000	58.7

**Table 2:** Summary of fatigue data for 2024-T4 specimens with a tapered notch [10].

Stress Amplitude, Δσ (MPa)	Sample Size, *n*	Median Life	Sample Average, $\bar{x}$	Sample Standard Deviation, *s*	Sample Coefficient of Variation, *cv* (%)
216	21	15,700	16,200	1460	8.97
206	30	20,700	22,100	2020	9.13
176	30	47,100	49,900	4620	9.26
147	30	128,000	134,000	11,500	8.58
118	30	326,600	348,000	41,600	12.0
98	30	806,800	843,000	137,000	16.2
78	30	1,959,200	2,080,000	492,000	23.7
69	30	3,443,200	3,680,000	1,090,000	29.5
64	21	7,412,000	7,280,000 *	2,270,000 *	31.2 *

* indicates censored data.

**Table 3:** Weibull parameters used in the fatigue crack growth model.

Random Variable	α	β	γ	μ	*cv* (%)
initial damage size *a_o_* (m)	0.95	9.75 × 10^−6^	6.5 × 10^−6^	16.5 × 10^−6^	63.8
fatigue coefficient *C* (m/cyc)/(MPa√m)^3.33^	3.0	3.5 × 10^−12^	1.0 × 10^−12^	4.13 × 10^−12^	27.5
threshold driving force Δ*K_th_* (MPa√m)	8.75	0.30	0.25	0.53	7.3

## Data Availability

The original contributions presented in the study are included in the article, further inquiries can be directed to the corresponding author.
